# Proactive Personality and Turnover Intention: The Mediating Role of Career Aspiration and the Moderating Effect of Organizational Career Management

**DOI:** 10.3390/bs14090800

**Published:** 2024-09-10

**Authors:** Qiong Wang, Zhe Shang, Chenhui Zuo, Huaye Fan, Chen Xu, Zijun Cai, Wei Shi

**Affiliations:** 1 College of Business, Beijing Open University, Beijing 100081, China; wangqiong@bjou.edu.cn; 2 School of Government, Beijing Normal University, Beijing 100875, China; zshang@bnu.edu.cn (Z.S.); zuochenhui@mail.bnu.edu.cn (C.Z.); 3 Business School, Beijing Normal University, Beijing 100875, China; 202311030026@mail.bnu.edu.cn (H.F.); 202111030009@mail.bnu.edu.cn (C.X.); 4 School of Labor and Economics, Remin University of China, Beijing 100875, China

**Keywords:** proactive personality, career aspirations, perceived organizational career management, turnover intention

## Abstract

As proactivity becomes vital for organizational success, retaining proactive employees becomes increasingly important, making the relationship between a proactive personality and turnover intention a key research topic. While existing studies have largely depicted turnover as a consequence of dissatisfaction and have identified negative indirect relationships, this study seeks to challenge that perspective by proposing that, in today’s boundaryless career environment, people also engage in voluntary turnover for career advancement. Using a self-regulation career management model, we propose that proactive employees set ambitious career goals influenced by career aspirations, leading them to seek external opportunities and thus exhibit higher turnover intention. However, when organizations implement career management practices, this relationship weakens as proactive employees perceive opportunities to achieve their goals within their current organizations. We tested these hypotheses with a sample of 342 respondents using the SPSS macro PROCESS. The findings support our propositions, revealing a positive indirect effect through career aspirations, which diminishes when perceived organizational career management is strong.

## 1. Introduction

Organizations rely heavily on their employees’ proactivity to survive and succeed in the current uncertain and competitive environment, thus placing high importance on attracting and retaining proactive employees [1,2]. However, the relationship between a proactive personality, i.e., the behavioral tendency to impact and manipulate environments through creating changes [3], and turnover intention has not received enough attention. The limited existing research primarily highlights a negative relationship between a proactive personality and turnover intention, suggesting that proactive individuals create favorable work environments by, for instance, managing work demands [4], displaying less surface acting [5], and building more trusting interpersonal relationships [6]. The rationale is that a better work environment increases job satisfaction among proactive employees, thereby reducing their intention to leave. 

However, the studies ignored the fact that the decision to stay or leave also involves career considerations. In the era of boundaryless careers, people need to frequently move across organizations to facilitate vocational development [7,8]. Besides avoiding dissatisfying working experiences, people might leave their current organizations to seek better career prospects [9,10]. Since proactive people actively manage their careers [11,12], the relationship between a proactive personality and turnover intention might differ when viewed from this career-oriented perspective—a point largely neglected in previous research. 

This study aims to fill the gap. Drawing on a self-regulation career management model [13,14,15], we propose that proactive people set high career goals, as reflected by high career aspirations [16], because they are naturally inclined to make improvements and create differences [3], especially in their careers. High career aspirations motivate people to take action, and one strategy is to approach external employment opportunities, which increases their turnover intention [9]. Thus, career aspirations might mediate the relationship between a proactive personality and turnover intention. We further argue that when proactive people perceive that they could achieve their aspirations in their current organizations, they are less likely to leave. Correspondingly, we propose that when perceived organizational career management, which refers to the perceptions of management practices to facilitate career development [17], is high, the relationship between career aspirations and turnover intention, and thus the indirect relationship, is weaker.

**Figure 1:**
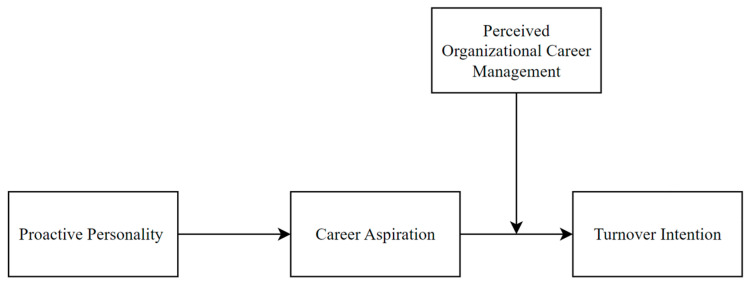
Theoretical model. This figure describes the theoretical model we propose and aim to examine in this study: a proactive personality might influence turnover intention through career aspirations, with this indirect relationship contingent on perceived organizational career management.

In short, we propose and examine a second-stage moderated mediation model in this study (see Figure 1). By doing so, we can make at least three contributions. First, we provide a new perspective to understand the relationship between a proactive personality and turnover intention. We show that career experiences could also explain why proactive people want to leave. Second, we enrich the understanding of how organizational management practices influence proactive people. Previous studies mainly focused on the influences of leader- and team-related factors. We show that organizational career management could help retain proactive people. Third, although not our main focus, we show a new individual factor, i.e., career aspirations, that influences the effectiveness of perceived organizational career management (POCM), which responds to the call to further examine the boundaries of POCM [18].

## 2. Literature Review and Hypotheses Development

### 2.1. Proactive Personality and Existing Studies about Its Relationship with Turnover Intention

A proactive personality reflects one’s dispositional tendency to take initiative despite external influences. It is characterized by a keen awareness of areas for improvement, strong confidence in effecting change, optimism about outcomes, and a persistent drive to bring about change [3]. Consequently, individuals with a proactive personality are typically those who actively seek opportunities, take the initiative, follow through with actions, and persevere until they achieve their desired outcomes [3]. 

Research on the relationship between a proactive personality and turnover intention has predominantly focused on how this personality trait manifests in the workplace. These studies suggest that proactive individuals create more favorable work environments [19], which increases their job satisfaction and encourages them to remain with their current employers. For instance, social capital has been identified as a mediator in the negative relationship between a proactive personality and turnover intention, suggesting that proactive people cultivate a supportive social environment [6]. Similarly, surface acting has been found to mediate this relationship [5], with the argument being that proactive individuals are less constrained by organizational norms and rules, resulting in reduced surface acting. This reduction in surface acting leads to less exhaustion, making proactive employees less dissatisfied and, therefore, less likely to leave. Family–work interference has also been found to mediate the negative relationship, as proactive employees tend to focus their resources on work demands [4]. With fewer interferences, these employees are less inclined to avoid working in their organizations.

Generally, these studies see turnover as a dissatisfaction-driven process: when people feel happy at work, they are less likely to leave; however, when they are dissatisfied, they are motivated to avoid such experiences and thus intend to leave [20]. These studies overlook the fact that, as careers become more boundaryless, individuals may proactively change employers in pursuit of better career opportunities [7,8]. A proactive personality can also be expressed in career management and can influence career goals and corresponding behaviors [21]. From a career-focused perspective, this suggests that the relationship between a proactive personality and turnover intention may differ from the conventional view, as we will further explore.

### 2.2. Understanding Turnover Intention from a Career-Focus Perspective

In the era of boundaryless careers, career patterns have become more unpredictable and non-linear, and people have more autonomy and responsibility to manage their careers [22]. Articulating people’s agency, scholars argued that career management could be seen as a goal-directed self-regulation process [23,24,25]. In this process, both personal factors, such as personality traits, and contextual factors, such as labor market conditions, influence career goals by shaping motivation, efficacy beliefs, and emotions. These goals, in turn, drive individuals to take actions aimed at reducing the gap between their current situation and their desired status. Based on feedback, individuals continuously adjust their goals, plans, and actions. Through repeated cycles of this process, individuals build their careers. 

Scholars have identified many behaviors to narrow the gap and achieve career goals, such as exploring self and environment in-depth, socializing in new companies, and trying new ways to finish work. Voluntary turnover becomes a potential strategy when individuals feel that their current organization no longer aligns with their ambitions and aspirations [25]. In pursuit of further growth and better opportunities, individuals may choose to leave their current organization and seek employment elsewhere [9]. Indeed, scholars have seen mobility willingness and actions as necessary for career success in the era of boundaryless careers [10]. 

In short, from the career-focused perspective, particularly within the self-regulation career management model, turnover intention can be seen as a reflection of an individual’s intention to actively reduce the gap between their goals and current status by changing jobs. In this context, a proactive personality may influence turnover intention through this goal-driven self-regulation process. 

### 2.3. Proactive Personality, Career Aspirations, and Turnover Intention

According to the self-regulation career management model, personal factors influence behavioral decisions through the process of goal setting [13]. Individuals with a highly proactive personality possess an intrinsic drive to change their current circumstances. They also have a strong belief in their ability to overcome obstacles and effect change [3]. As a result, they are likely to set ambitious and challenging career goals, motivated by a desire to improve their career prospects and a belief in their ability to achieve these goals. Career aspirations refer to the expressed career-related goals or ambitions [16]. High career aspirations set challenging goals, such as becoming a leader in the career field and continuously moving up. Accordingly, a proactive personality should be positively related to career aspirations. 

The self-regulation career management model suggests that goals direct individuals to change their behaviors. High career aspirations indicate significant discrepancies between desired future outcomes and the current situation, motivating individuals to take action to close this gap. One key strategy for achieving this is mobility-oriented behavior, which involves leaving the current organization in search of better opportunities. [26]. This is particularly relevant in the boundaryless career world, characterized by job insecurities and career uncertainties, where individuals need to seek and seize opportunities within and outside current organizations simultaneously [7,10]. As a result, to achieve their aspirations, proactive individuals may actively pursue external opportunities, leading them to consider turnover as a viable option [9]. Indeed, previous studies have explicitly noted that “employees may voluntarily leave an organization in order to pursue desirable career opportunities at another organization … if they believe it is the best way to accomplish their career goals” [27]. Therefore, proactive individuals may develop a stronger intention to leave. Additionally, even if proactive individuals employ other strategies to close the gap, such as building networks and developing skills [22], these would still increase the possibility of receiving unsolicited job offers, therefore raising turnover intention [9].

In summary, proactive individuals are likely to set high career goals, as reflected by elevated career aspirations. These high aspirations drive individuals to take action, with one option being to leave their current organizations for better opportunities, resulting in higher turnover intention. Thus, we propose the following:

**Hypothesis 1:**  *Career aspirations mediate the positive relationship between a proactive personality and turnover intention*.

### 2.4. The Moderation Role of POCM 

Self-regulation models emphasize the role of contextual factors in influencing the relationships between goals and behaviors [28,29]. In the context of career management and voluntary turnover, organizational practices are believed to play a crucial role in how individuals translate their goals into behavioral decisions [9,15]. The arguments presented earlier suggest that the positive indirect relationship between a proactive personality and turnover intention hinges on proactive individuals seeking external opportunities to fulfill their aspirations. However, when individuals believe they can achieve their aspirations within their current organizations, they may perceive moving to another organization as less attractive, leading to lower turnover intention. Accordingly, we propose that POCM, which reflects people’s perception of organizational support for their career development [18], be a moderator.

Organizational career management includes a set of practices that aim to facilitate employees’ career development within current organizations [17]. Typical practices include training, mentoring, and job rotation. When people perceive that their organizations provide such practices, they are more likely to feel that organizations care and support their career development [30]. In these organizations, people could get rich resources and opportunities to develop their careers, which are critical to narrowing the gap between desired future career status and current situations [18]. 

When POCM is high, individuals with strong career aspirations feel that they have ample opportunities to achieve their goals. For example, when they want to be a leader in their career fields, the organizations’ training, mentoring, outplacement, and clear career ladders help them get the necessary resources and set them on the paths to reach the positions. In this context, remaining with the current organization becomes a desirable option due to the potential for goal (career aspirations) fulfillment. Indeed, previous studies have shown that the alignment between career support and career goals is a significant motivator for employees to dedicate their efforts to their current organization [27]. This alignment fosters a sense of fulfillment of psychological contracts [31], which makes them less likely to leave. By contrast, when POCM is low, individuals might feel that they cannot reach their aspirations in their current organizations. They are more likely to search for external opportunities, attracted by alternative employment, and thus have stronger turnover intentions. Thus, we propose the following:

**Hypothesis 2:**  *POCM moderates the relationship between career aspirations and turnover intention, such that the relationship becomes weaker when POCM is higher*.

### 2.5. The Moderated Mediation Model 

Based on the self-regulation career management model and the preceding arguments, proactive individuals are likely to have high career aspirations. Whether these aspirations lead them to consider leaving their organization may depend on the organizational career management practices in place. When proactive individuals perceive strong career support from their organization, they believe their aspirations can be fulfilled within the organization. Driven by their natural motivation to close the gap between their current situation and their desired state (as reflected in their career aspirations), they are less likely to leave their current employer. Conversely, when these individuals perceive a lack of career management practices in their organization, they may realize that achieving their aspirations within the organization is unlikely. Consequently, they might actively seek external opportunities, leading to a higher turnover intention. Taking all these into consideration, we propose the following:

**Hypothesis 3:**  *POCM moderates the indirect relationship between a proactive personality and turnover intention through career aspirations, such that the relationship becomes weaker when POCM is higher*.

## 3. Method

### 3.1. Sample and Procedures

Data used in this study were collected from a large company in Beijing, China, chosen for its diverse functions and locations, which ensured a varied sample. We directly contacted the CEO and gained his approval for the research. We approached employees through WeChat, the most popular social app in China. Specifically, we provided the HR department with a link containing details about our research, which they then distributed to all employees in the company. The link explained that the study aimed to explore the relationship between personality and work behavior. We assured participants that their data would be used exclusively for research purposes, stored securely on the research team’s computer, and accessible only to team members. We also clarified that no identifiable information would be disclosed in any publications, with only general findings and relationships being reported. Interested employees could join a WeChat group by scanning a QR code, through which we distributed links to the online surveys.

To relieve the concern about common method bias, we collected data from two different time points. At time one, demographic information and a proactive personality were collected. After four weeks, career aspirations, POCM, and turnover intention were collected. The data from both time points were matched using the last four digits of the participants’ ID cards, which they could choose to report or not. If they did not, their answers were dropped. 

At time one, we got 425 respondents. At time two, we got 353 respondents. After dropping the missing data, we got 342 matched samples. Most participants were male (N = 255, 74.13%). Seven graduated from primary school. Twenty-four graduated from high school. Fifty-five graduated from college. Two hundred fifty-four hold a bachelor’s degree. Two hold a master’s degree. Their average age was 35.91 years old (SD = 10.30 years old). They were from different departments and project teams, such as human resource management, accounting, construction, IT, marketing, and law. They also did various jobs, such as sales, construction workers, security, accounting, clerks, QC, and HR. This diversity strengthens the validity of our analyses.

### 3.2. Measurements

We used a 5-point Likert scale to measure all variables. People were asked to rate their agreement with the items from 1 (extremely disagree) to 5 (extremely agree). To ensure measurement reliability and validity, we selected formally published scales that were demonstrated to effectively measure the corresponding construct. When scales were previously adopted in China, we used that version. Otherwise, we went through several rounds of translation and back-translation until we reached a final consensus. The full scales are attached in Appendix A.

Proactive personality was measured by the ten-item short scale [12]. A sample item is “I am constantly on the lookout for new ways to improve my life”. In this study, Cronbach’s alpha is 0.87.

Career aspirations were measured by the five-item scale [22]. A sample item is “I hope to become a leader in my career field”. In this study, Cronbach’s alpha is 0.90.

POCM was measured by the eleven-item scale adopted from a previous study in the Chinese context [17], which measured to what extent people perceive their organizations provided the mentioned practices, such as mentoring programs, succession planning, and outplacement. In this study, Cronbach’s alpha is 0.96.

Turnover intention was measured by the four-item scale [32]. A sample item is “I am thinking about leaving this organization”. In this study, Cronbach’s alpha is 0.93.

We initially planned to control for demographic variables in our analyses. However, the results remained consistent regardless of whether these variables were included. Therefore, for the sake of brevity, we present the results without including demographic controls. 

Additionally, we conducted one-way ANOVA analyses and found that none of the variables showed significant variance between departments or job roles. Consequently, there was no need to account for differences related to department or job type in our analyses [33]. 

## 4. Results

### 4.1. Confirmatory Factor Analyses

We conducted confirmatory factor analyses to examine the measurement validity using Mplus. The results are presented in Table 1. As we can see, the hypothesized four-factor model fits the data best: χ2 = 1200.52, df = 399, RMSEA = 0.08, CFI = 0.90, and SRMR = 0.06.

### 4.2. Descriptive Statistics and Simple Correlations 

Descriptive statistics and simple correlations are presented in Table 2. The results showed that a proactive personality was positively related to career aspirations (r = 0.23, *p* < 0.001), which were positively related to turnover intention (r = 0.25, *p* < 0.001). These results suggest a possible mediation effect.

### 4.3. Mediation Effect Analyses 

We used SPSS to run the linear regressions to test the mediation effect (Hypothesis 1). Model 4 in SPSS macro PROCESS was used. A proactive personality and career aspirations were mean centered before analyses. All reported 95% confidence intervals (CIs) were calculated using a bias-corrected bootstrapping method with 5000 bootstrap samples.

The results are shown in Table 3. As we can see, a proactive personality was positively related to career aspirations (β = 0.40, *p* < 0.001). Career aspirations were positively related to turnover intention (β = 0.30, *p* < 0.001). The indirect effect was significant: effect = 0.12, 95% CI = [0.05, 0.22]. The direct effect was not significant (β = −0.06, n.s.). Thus, Hypothesis 1 passed the examination. 

### 4.4. Moderation Effect Analyses

Model 1 in SPSS macro PROCESS was used to examine the moderation effect (Hypothesis 2). Career aspirations were mean centered before analyses. Similar to the above, we calculated the 95% CI using the bias-corrected bootstrapping method with 5000 bootstrap samples. We controlled proactive personality, according to the suggestion about how to examine the moderation effect in a second-stage moderated mediation model [34].

**Figure 2:**
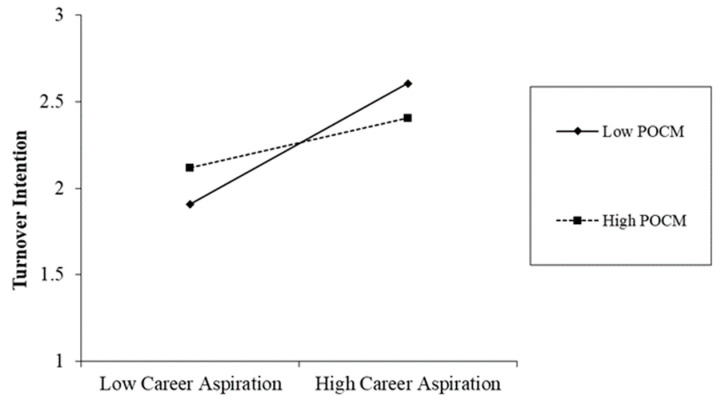
Simple slope analysis results.

From Table 3, we can see that POCM significantly moderated the relationship between career aspirations and turnover intention (β = −0.12, *p* < 0.05). Simple slope analysis showed that when POCM was at the higher level (+1 SD), the relationship between career aspirations and turnover intention was not significant (β = 0.16, n.s.). When it was at the lower level (−1 SD), the relationship was stronger and significantly positive (β = 0.40, *p* < 0.001). We drew Figure 2 to present the results. Thus, Hypothesis 2 passed the examination. 

### 4.5. Moderated Mediation Effect Analyses

**Figure 3:**
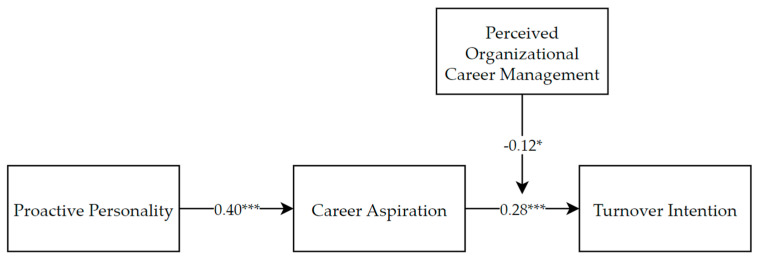
Moderated Mediation Examination Results. * *p* < 0.05; *** *p* < 0.001.

Finally, Model 14 in SPSS macro PROCESS was used to examine the moderation effect. The results are displayed in Figure 3. Again, 95% CI was calculated with the bias-corrected bootstrapping method based on 5000 bootstrap samples. The results showed that the moderated mediation index was significant: index = −0.05, 95% CI = [−0.11, −0.002]. When POCM was at the higher level (+1 SD), the indirect effect was not significant (effect = 0.06, 95% CI = [−0.03, 0.17]). When it was at the lower level (−1 SD), the indirect effect was significant (effect = 0.16, 95% CI = [0.06, 0.28]). Thus, Hypothesis 3 passed the examination. 

### 4.6. Supplementary Analyses

Some might argue that according to trait activation theory, POCM might moderate the relationship between a proactive personality and career aspirations. Analyses showed a non-significant effect (β = 0.09, n.s.). The reason might be that a proactive personality reflects a tendency to shape the environment, so it might compensate for the lack of organizational career management practices [35]. The two co-existing forces together led to the non-significant effect. 

## 5. Discussion

In this study, we propose that, to advance their careers, proactive people might develop high turnover intention. In detail, we argue that proactive people might have high career aspirations, which are positively associated with turnover intention. However, when POCM is high, these people would feel that they can achieve their aspirations in their current organization and thus have low turnover intention. Analysis showed that this second-stage moderated mediation model was supported. The results have important theoretical and practical implications. 

### 5.1. Theoretical Implication

This study offers a new perspective for understanding proactive employees’ voluntary turnover. Existing studies mainly focus on their work experiences [36]. These studies imply that proactive people intend to leave when they feel bad about their work. We take a different perspective by considering turnover intention a career issue and argue that proactive employees might intend to leave their organizations to pursue their career aspirations [25]. To the best of our knowledge, hardly any studies have taken such a perspective. The only two exceptions treat proactive personality as a moderator [37,38]. They find that a proactive personality enhances the positive relationship between career commitment and turnover intention and compensates for the relationship between lack of providing mentoring experiences, career plateaus, and turnover intention. We advance their studies by directly focusing on the influence of a proactive personality and revealing a new mechanism: career aspirations. We show that when POCM was lower, the indirect effect was significant and stronger, indicating that proactive people intend to leave their current organizations to better strive for their career goals. The results should encourage scholars to further examine the role of career-related factors, such as employability and other career resources [39]. 

This study enriches the understanding of how to manage proactive people at work. A proactive personality has been linked to many favorable outcomes, such as high job performance and proactivity [19]. How to manage proactive people and increase their work effectiveness has thus become an important issue. Previous studies mainly focused on leader- and team-related factors. For example, from a fit perspective, scholars showed that proactive colleagues and value congruence are important to ensure proactive people’s output [40,41,42]. However, existing studies largely ignored the importance of organizational management practices. A growing body of studies began to examine how management practices influenced employee proactivity [43,44], but scholars have seldom examined how management practices would influence proactive employees [38]. We showed that career management practices could help retain these people because the practices provide resources and opportunities to achieve their career aspirations. Future studies could examine whether POCM could influence other outcomes. For example, following the same logic, proactive people might feel more gratitude when POCM is high, so they would display more pro-organizational behavior. 

This study also helps us better understand the boundary of the effect of POCM. Previous studies have shown that POCM could influence people in many ways, but only a few studies examined who would benefit more from this perception or the practices. Indeed, examining the boundary effects has been set as a key future direction [18]. In one of the limited studies, people with high career adaptability were found to develop lower turnover intention when POCM was high [17]. We extend their findings by showing that a proactive personality is also an important individual factor because of the high career aspirations it brings. Career adaptability, a proactive personality, and career aspirations all reflect the readiness to change one’s career situation. Future studies could examine whether other career-related individual factors could also influence the outcome of POCM. 

Notably, we found that a proactive personality was not significantly correlated with turnover intention. When controlling the effect of career aspirations, the direct effect was still not significant. In previous studies, some scholars also found insignificant correlations [38,45,46]. The results imply that a proactive personality serves as a pretty distal predictor of turnover intention. Another possibility is that there are many contrasting mechanisms. Even after considering the mediation effect of career aspirations, the existence of other contrasting mechanisms, such as different kinds of work-family conflict [4], still makes the left total effect zero. This indicates the importance of further examining other possible mechanisms. 

### 5.2. Practical Implication

There are two key takeaways for practitioners. First, while the benefits of employing proactive individuals are well-documented, leading to recommendations for using proactive personality scales during recruitment [47], the focus has often been more on hiring rather than managing these employees. Previous studies have suggested that proactive employees are less likely to leave because they create favorable work environments. However, our study reveals an often-overlooked risk: proactive employees may have a strong intention to leave due to their high career aspirations. Therefore, it is essential for organizations to shift their managerial approach. This can be achieved through training sessions that help managers identify and understand the aspirations of proactive employees. Regular, open-ended career development discussions, where managers actively listen to employees’ goals and ambitions, are crucial. Tools like career aspirations assessments can be used to systematically capture and analyze the aspirations of proactive employees, providing insights into whether they might be considering leaving.

Moreover, to mitigate the turnover intentions of proactive employees, our findings suggest that organizations need to implement comprehensive career management strategies that support these employees in achieving their career aspirations. When proactive employees believe they can fulfill their career goals within the organization, they are less likely to consider leaving. For instance, organizations can work collaboratively with these employees to create personalized career plans that align their aspirations with the organization’s vision and objectives. This could involve designing clear career paths, offering internal mobility programs, providing mentoring, and supporting external training opportunities. Managers should be encouraged to act as career facilitators rather than just supervisors. They should establish clear, achievable milestones for proactive employees, conduct regular reviews and adjustments as employees progress, and provide continuous performance feedback. Additionally, they should assign these employees to key tasks, advise them on potential career opportunities, and act as their advocates within the organization. Ultimately, the goal is to ensure that proactive employees feel their aspirations can be realized within the organization.

### 5.3. Limitations and Future Directions

Despite the interesting findings, future studies could improve this study in several ways. First, although we provide a new perspective by focusing on career development, we did not compare the mediation effect of career aspirations with other mechanisms, such as work-family conflict. Future studies could examine whether this new perspective had incremental explanatory power. Second, the nature of this study is correlational. To better examine the effectiveness of career management practices, intervention-based studies are recommended [48]. Second, the correlational nature of this study limits our ability to draw causal conclusions. To better assess the effectiveness of career management practices, intervention-based studies are recommended. Additionally, from a constructivist perspective, it is possible that individuals with high turnover intention might retrospectively attribute their decision to career aspirations. Future research should consider the potential reciprocal relationship between these variables. To determine causality more accurately, future studies should employ intervention experiments, longitudinal designs, and instrumental variable techniques.

Third, we measured turnover intention but not actual turnover behavior. Although intention has been regarded as a strong indicator of actual behavior, research has shown that they are different [46]. Future studies could try to replicate our findings with actual behavior as the outcome. Fourth, our data were collected from a single Chinese company. To evaluate the generalizability of our findings, future research should test our model in different contexts to determine whether the results can be replicated. For instance, in individualistic cultures where career development is more self-driven, would the mediating effect of career aspirations be stronger? Would the importance of organizational career management diminish? Additionally, most of our participants were well-educated, with bachelor’s degrees. For less educated individuals who typically face a more challenging labor market, would they develop lower career aspirations and turnover intentions, even if they are proactive?

## 6. Conclusions

Existing studies on the relationship between a proactive personality and turnover intention predominantly highlight a negative indirect effect, often explained through a dissatisfaction-driven process. This study challenges that perspective by adopting a career-focused approach. Drawing on a self-regulation career management model, we showed that proactive individuals’ high career aspirations lead them to consider leaving their organizations unless their organizations provide robust career management practices. The findings suggest that scholars should explore this relationship from alternative perspectives, and they also underscore the importance of organizations developing effective strategies to retain proactive employees, who are essential to their survival and competitiveness.

## Figures and Tables

**Table 1:** Confirmatory factor analyses results.

Model	χ^2^	df	Δχ2/df	RMSEA	CFI	SRMR
Hypothesized 4-factor	1200.52	399	/	0.08	0.90	0.06
3-factor (PAP+CP, POCM, TU)	2139.43	402	312.97 ***	12	0.78	0.12
2-factor (PAP+CP+TU, POCM)	3241.73	404	551.15 ***	0.14	0.62	0.17
1-factor	6870.27	405	3628.54 ***	0.17	0.22	0.28

PAP = proactive personality, CP = career aspirations, POCM = perceived career organizational management, TU = turnover intention; “+” means that the variables were combined. *** *p* < 0.001.

**Table 2:** Descriptive statistics, reliability coefficients, and inter-correlations.

	Mean	SD	1	2	3	4
1. Proactive personality	3.72	0.51	**0.87**			
2. Career aspirations	3.20	0.88	0.23 ***	**0.90**		
3. Turnover intention	2.24	1.04	0.03	0.25 ***	**0.96**	
4. Perceived organizational career management	2.78	0.97	0.09	0.27 ***	0.05	**0.93**

Reliability coefficients, i.e., Cronbach’s alpha coefficients, were shown along the diagonal in bold. *** *p* < 0.001.

**Table 3:** Regression analyses results.

	Career Aspirations	Turnover Intention	
	Model 1	Model 2	Model 3
Proactive personality	0.40 ***	−0.06	−0.04
Career aspirations		0.30 ***	0.28 ***
POCM			0.002
Career aspiration × POCM			−0.12 *

POCM = perceived organizational career management. * *p* < 0.05. *** *p* < 0.001.

## Data Availability

Data available on request due to restriction.

## Appendix A

The scales we used in this study are as follows:

Proactive personality [12]:

No matter what the odds, if I believe in something, I will make it happen.

I love being a champion for my ideas, even against others’ opposition.

I excel at identifying opportunities.

If I believe in an idea, no obstacle will prevent me from making it happen.

I am constantly on the lookout for new ways to improve my life.

Wherever I have been, I have been a powerful force for constructive changes.

Nothing is more exciting than seeing my ideas turn into reality.

If I see something I do not like, I fix it.

I am always looking for better ways to do things.

I can spot a good opportunity long before others can.

Career aspirations [22]:

I hope to become a leader in my career field.

When I am established in my career, I would like to manage other employees.

When I am established in my career, I would like to train others.

I hope to move up through any organization or business I work in.

I think I would like to pursue graduate training in my occupational area of interest.

Turnover intention [32]:

I am thinking about leaving this organization.

I am planning to look for a new job.

I intend to ask people about new job opportunities.

I do not plan to be in this organization much longer.

Perceived organizational career management [17]:

(to what extent you perceive the organizations provide the following practices)

mentoring programs, succession planning, outplacement, career ladders and paths, job posting, individual counseling, external training opportunities, in-house training, promotability forecasting, job rotation, and developmental assessment centers.
